# Effects of intensive insulin therapy on the retinal microvasculature in patients with type 2 diabetes mellitus: a prospective observational study

**DOI:** 10.1186/s12886-022-02397-9

**Published:** 2022-04-22

**Authors:** Ning Yang, Ming-Xin Li, Xiao-Yan Peng

**Affiliations:** 1 Department of Ophthalmology, Beijing Tongren Hospital, Capital Medical University, Beijing Institute of Ophthalmology, Beijing Tongren Eye Center, Beijing Ophthalmology and Visual Science Key Laboratory, No.17 Hougou Lane, Chongnei Street, Beijing, 100005 China; 2 Department of Ophthalmology, The Affiliated Hospital of Xuzhou Medical University, Quanshan District, 99 West Huaihai RdJiangsu, Xuzhou, 221002 China

**Keywords:** Retinal microvasculature, Intensive insulin therapy, Optical coherence tomography angiography

## Abstract

**Background:**

We examined the retinal microvascular changes and associated factors in type 2 diabetes mellitus (T2DM) before and after intensive insulin therapy.

**Methods:**

This prospective observational study recruited patients with T2DM and divided them into intensive insulin therapy and oral hypoglycemic agent groups. All patients enrolled in this study had diabetes without retinopathy or non-proliferative diabetic retinopathy. Optical coherence tomography angiography (OCTA) was used in all patients before treatment and at 1, 3, and 6 months after treatment. Vessel density (VD) and thickness changes in the macular and optic disc areas were assessed.

**Results:**

The study included 36 eyes in the intensive insulin therapy group and 36 in the oral hypoglycemic agent group. One month after treatment, VD in the deep capillary plexus (DCP) and peripapillary capillary VD (ppVD) were significantly decreased by intensification (*P* = 0.009, 0.000). At three months after treatment, decreases in VD induced by intensification were found in the superficial capillary plexus (SCP), DCP, foveal density in a 300-μm-wide region around the foveal avascular area (FD-300), and ppVD (*P* = 0.032, 0.000, 0.039, 0.000). Six months after treatment, decreases in VD by intensification were observed in the DCP and ppVD groups (*P* = 0.000, 0.000). Vessel density showed no significant change in the oral hypoglycemic agent group after treatment. The amount of DCP-VD reduction was correlated with macular thickening (*r *= 0.348, *P* = 0.038; *r* = 0.693, *P* = 0.000 and *r* = 0.417, *P* = 0.011, respectively) after intensive insulin therapy.

**Conclusions:**

Insulin-intensive treatment caused a transient reduction in vessel density in the macular and optic disc areas. DCP-VD and ppVD were more susceptible at an earlier stage. Retinal microvasculature monitoring using OCTA is vital for patients with type 2 diabetes receiving intensive insulin therapy.

**Supplementary Information:**

The online version contains supplementary material available at 10.1186/s12886-022-02397-9.

## Background

Diabetic retinopathy (DR) is the most common microvascular complication of diabetes mellitus and one of the significant causes of blindness in the working-age population [1]. Chronic high blood glucose levels damage the retinal capillaries and nerves, playing an essential role in the occurrence and development of DR [2]. Short-term intensive insulin therapy is very important in type 2 diabetes mellitus, which was given by either continuous subcutaneous insulin infusion or multiple daily injections for 14–21 days [3]. Many population-based clinical studies have shown that intensive insulin therapy in patients with type 2 diabetes can help normalize blood glucose control in the short term, thus delaying the progression of DR [4, 5]. However, some studies have indicated that intensive insulin therapy can aggravate diabetic retinopathy at an early stage [6, 7]. The pathophysiology of this phenomenon is not well known, although the existence of early worsening of various diabetic therapies is not in doubt [8]. Several possible mechanisms [9] have been summarized to explain this phenomenon, including the co-synergistic effects of insulin and vascular endothelial growth factor (VEGF) on retinal capillaries, the blood-retinal barrier breakdown speculation, and upregulation of VEGF theory, all of which remain tentative and inconclusive. Previous studies have focused on the progression of diabetic retinopathy severity scale and clinically significant diabetic macular edema (DME). However, there is no information on retinal vessel density and the changes in the different layers of the retinal vascular plexus following intensive insulin therapy. Optical coherence tomography angiography (OCTA) can quantify retinal vessel density and thickness changes, and has been widely used to evaluate retinal ischemic diseases [10] and neuroophthalmological diseases [11]. With the development of OCTA, macular microvasculature in DR, including the superficial and deep capillary plexus, can be quantified noninvasively and repeatedly [12]. The current OCTA study focused on type 2 diabetic patients and aimed to study how their retinal microvasculature networks responded to intensive insulin therapy, compared with the oral hypoglycemic agent group. We also aimed to evaluate the association between parameters of vessel density, FAZ area, and thickness of the macular area at different time points in patients with insulin intensification.

## Methods

This prospective study was conducted following the tenets of the Declaration of Helsinki. The ethics committee of the Affiliated Hospital of Xuzhou Medical University approved the study protocol (No. XYFY2019-KL071-01). All participants provided written informed consent.

### Subjects and clinical protocols

This study enrolled 77 patients with T2DM from May 2020 to February 2021. Fasting blood glucose (FBG) was ≥ 11.1 mmol/L or HbA1c ≥ 9.0%. The diagnosis of T2DM was made by endocrinologists (Dr. Hong-Wei Ling, Dr. Meng Zhao, Dr. Chang-Jiang Ying, et al.) according to the 2014 American Diabetes Association diagnostic criteria [13]. Clinical data, including a history of hypertension, hyperlipidemia, smoking, and renal impairment, were recorded using a standardized clinical record form [14]. In our research, hypertension was defined as a blood pressure greater than 140/90 mmHg, or a self-reported history of hypertension. Hyperlipidemia was defined as either a total cholesterol level of 6.2 mmol/L or the use of lipid-lowering drugs. According to different hypoglycemic methods, 39 patients were randomized into the intensive insulin group, and 38 patients were randomized into the oral hypoglycemic agent group. The diagnosis and classification of DR were confirmed according to the international clinical diabetic retinopathy and diabetic macular oedema disease severity scales [15]. One eye of each participant was included in the research. For patients without clinical DR (NDR), one eye was randomly selected. For patients with non-proliferative diabetic retinopathy (NPDR), the eye with a higher stage was set. If both eyes were in the same scene, one eye was randomly selected. Patients were excluded if they had any of the following conditions at baseline: 1) proliferative DR or diabetic macular edema; 2) diseases of the vitreoretinal interface and optic nerve; 3) other ocular diseases, including but not limited to age-related macular degeneration, retinal arteriovenous occlusion, retinal choroiditis, uveitis, hereditary macular disease, and other fundus diseases that may lead to structural and morphological changes of the macula; 4) a history of ocular trauma or intraocular surgery; 5) myopia more than -6.00 diopters or axial length of over 26.50 mm; and 6) failure to obtain high-quality images due to refractive interstitial opacity or poor coordination.

Short-term intensive insulin therapy involves injecting insulin at least three times daily (fast-acting insulin at three meals + long-acting basal insulin at bedtime. Insulin lispro or insulin aspart was the fast-acting insulins used. Insulin glargine, insulin detemir, or insulin degludec was the long-acting basal insulin used. Initial total daily insulin doses were 0.4–0.5 IU/kg, 40%-60% of which was long-acting basal insulin, and remained part was divided into 20%–40%–40% at three meals) or using continuous subcutaneous insulin infusion (Insulin lispro or insulin aspart. Initial total daily insulin doses were 0·4–0·6 IU/kg). The insulin dosage was adjusted based on the adequate frequency and scientific monitoring of blood glucose levels. In the group treated with oral hypoglycemic agents, patients typically received gliclazide or metformin twice a day. A combination of gliclazide and metformin was used in patients who could not reach the glycemic goal. After blood glucose stabilized, the patients were followed up weekly by telephone. Dosage was adjusted to maintain FBG of 4.4–7.0 mmol/L and non-FBG < 10.0 mmol/L [16]. HbA1c levels were monitored every three months. All enrolled patients underwent a comprehensive ocular examination, including best-corrected visual acuity by automatic refractor (KR-1, Topcon, Japan), intraocular pressure by noncontact tonometry (TX-20, Canon, Japan), slit-lamp microscopy evaluations of the anterior segment, dilated fundoscopic examination by binocular indirect ophthalmoscopy, and OCTA examination, before treatment and at 1, 3, and 6 months after blood glucose stabilization. OCTA was used to scan the macular area of the 6 × 6-mm mode and the optic disc area of the 4.5 × 4.5-mm mode in the two groups.

### OCTA image acquisition and analysis

OCTA scans were performed on all subjects using the Avanti RTVue XR system Quantization 2.0 (Version 2017.1, Optovue Inc. USA). The device uses a split-spectrum amplitude-decorrelation angiography algorithm with a three-dimensional projection artifact removal technique. All enrolled patients underwent OCTA examination in a dark room after pupillary dilation. The vessel density of the macular area was collected in the macular HD 6.0 × 6.0 mm scanning mode, and the optic disc area was collected in the HD disc 4.5 × 4.5 mm scanning mode. A single OCTA image acquisition consisted of a horizontal scan and a vertical scan to remove eye movement artifacts. The superficial capillary plexus (SCP) is defined as a slab extending from the internal limiting membrane (ILM) to 10 μm above the inner plexiform layer (IPL). The DCP is a slab extending 10 μm above the IPL to 10 μm below the outer plexiform layer (OPL). Foveal density in a 300 μm region around the foveal avascular zone (FD-300) is a parameter demonstrating capillary density from the ILM to the OPL in a 300 μm wide region around the foveal avascular zone (FAZ). Peripapillary vessel density (ppVD) and retinal nerve fiber layer (RNFL) thickness were quantified in the radial peripapillary capillary segment, defined as a slab extending from the ILM to the RNFL. The peripapillary area was defined as the area between annular contour lines of 2 mm and 4 mm around the disc margin. Vessel density was quantified using the split-spectrum amplitude decorrelation angiography software algorithm. The vessel density is defined as the percentage of signal-positive pixels per total pixel.

To ensure adequate scan quality and comparability, the OCTA scans required an image quality score of ≥ 6. All images were acquired by a single investigator (NY). The segmentation results of OCTA were manually checked and corrected if the boundary deviated from the right position. The following measurement parameters were quantified: vessel density of the SCP and DCP, the corresponding thickness of fovea and macula, FAZ area, FD-300, ppVD, and pRNFL thickness.

### Statistical Analysis

IBM SPSS Statistics (IBM Corporation, Chicago, IL, USA) for Windows (version 26.0) was used for statistical analysis, GraphPad Prism® (GraphPad Software Inc., La Jolla, CA) version 9.0.0 was used to plot graphs, and the Kolmogorov–Smirnov test was used to assess variable normality. One-way ANOVA and Chi-square tests were used to compare baseline clinical data between the two groups. Quantitative data are expressed as mean ± standard deviation ($\overline{x }$±s). Repeated measures ANOVA was used for changes in quantitative parameters at baseline and at 1, 3, and 6 months after treatment. Bonferroni’s post hoc test was used for correction, and comparisons between groups were performed using the independent samples t-test. Pearson correlation analysis was used to assess the association between parameters of vessel density, FAZ area, and thickness of the macula. Statistical significance was set at *P*0.05.

## Results

### General information

The study enrolled 77 patients with type 2 diabetes, excluding five patients (three patients had poorly controlled blood glucose levels and two patients did not finish scheduled check-ups within the follow-up stages). Table 1 shows the two groups’ basic clinical data and blood glucose levels. Systemic risk factors, such as hypertension, hyperlipidemia, and smoking at baseline, were similar between the intensive insulin and oral hypoglycemic agent groups. Among the patients who reached glycemic targets, the progress of glucose control, represented by fasting plasma glucose level and HbA1c, did not significantly differ between the two groups during the follow-up period. There were no significant differences in general data at baseline (*P* > 0.05).

**Table 1:** Basic clinical data of subjects

Characteristics	Intensive Insulin (*n* = 36)	Oral Agent (*n *= 36)	*P* values
Age (years)	54.3 ± 5.8	55.5 ± 6.0	0.40^*^
Gender (M/F)	21/15	19/17	0.64^†^
Duration of diabetes (years)	3.5 ± 1.6	3.4 ± 1.9	0.78^*^
SBP (mmHg)	132.9 ± 12.8	134.1 ± 11.7	0.69^*^
DBP (mmHg)	84.8 ± 10.8	83.8 ± 8.7	0.68^*^
Hypertension	12 (33.3%)	15 (41.6%)	0.63^+^
FBG (mmol/L)			
Pre-treatment	11.2 ± 1.9	10.8 ± 1.5	0.33^*^
At 1 M follow-up	5.6 ± 0.8	5.5 ± 0.9	0.65^*^
At 3 M follow-up	5.5 ± 0.9	6.0 ± 1.0	0.06^*^
At 6 M follow-up	5.4 ± 1.0	5.7 ± 0.9	0.17^*^
HbA1c (%)			
Pre-treatment	9.7 ± 1.1	9.9 ± 1.1	0.60^*^
At 3 M follow-up	5.7 ± 0.8	6.0 ± 0.7	0.24^*^
At 6 M follow-up	6.0 ± 0.7	5.8 ± 0.6	0.25^*^
CREA (μmol/L)	67.9 ± 21.4	74.8 ± 21.4	0.18^*^
UREA (mmol/L)	5.5 ± 1.6	5.7 ± 1.8	0.52^*^
eGFR (ml/min)	105.9 ± 15.5	112.0 ± 12.4	0.07^*^
TG (mmol/L)	1.8 ± 0.8	1.6 ± 0.7	0.36^*^
CHOL (mmol/L)	5.3 ± 1.4	4.9 ± 1.1	0.11^*^
Hyperlipidemia	12 (33.3%)	8 (22.2%)	0.43^†^
Smoking	13 (36.1%)	10 (27.8%)	0.61^†^

Data are shown as mean ± standard deviation

*P values are from one-way ANOVA

^†^*P* values are from Pearson Chi-square test

*SBP* systolic blood pressure; *DBP* diastolic blood pressure; *FBG* fasting blood glucose; *HbA1c* glycated hemoglobulin; *eGFR* estimated glomerular filtration rate; *CHOL* Cholesterol; *TG* triglyceride; *NPDR* non-proliferative diabetic retinopathy

### Changes of vessel density and thickness in the macular area

**Fig. 1:**
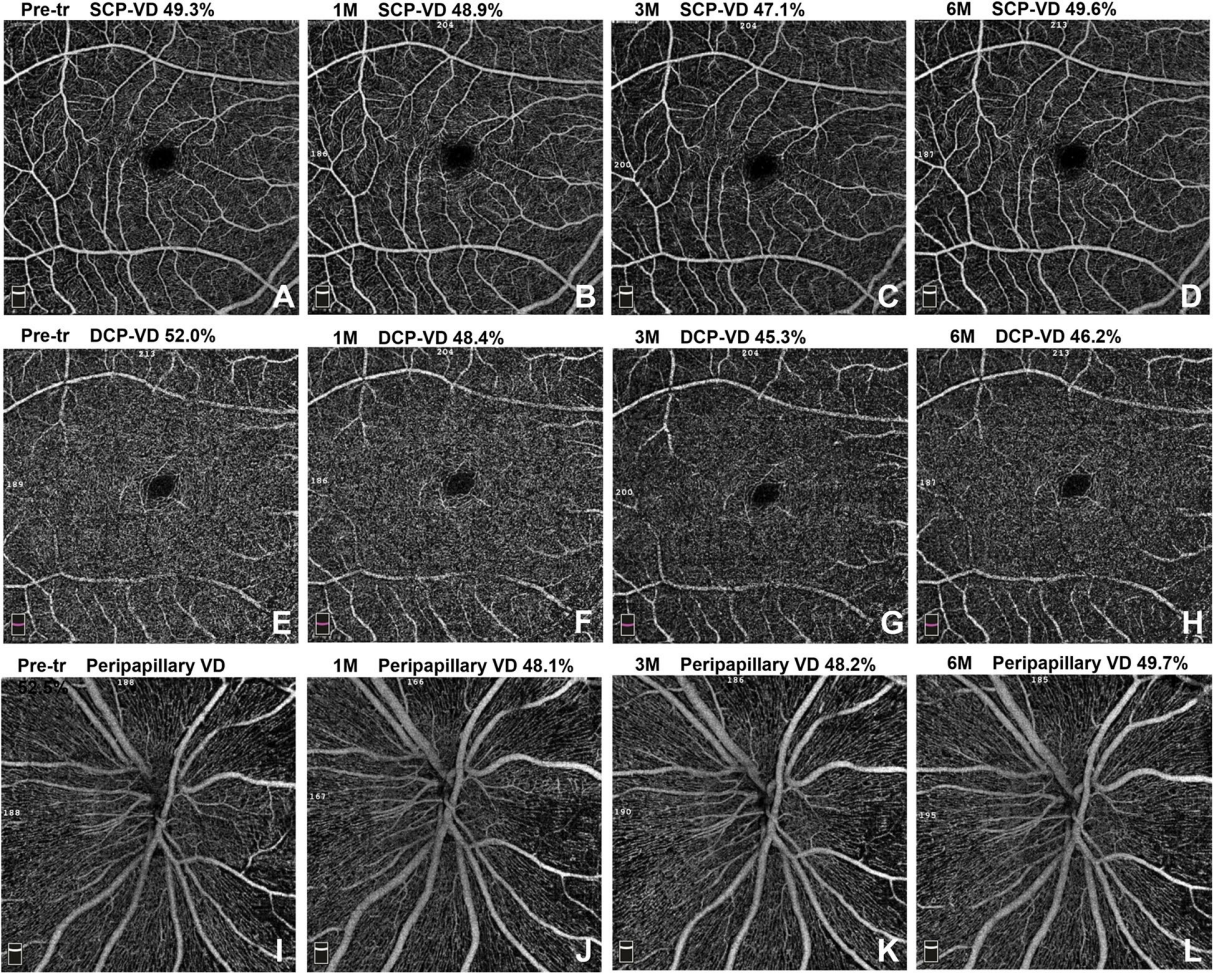
6.0 × 6.0 mm mode OCTA images of retinal vessel density changes in the intensive insulin group. **A ~ D** The superficial capillary plexus vessel density before and at 1,3,6 months after insulin intensification. **E ~ H** The deep capillary plexus vessel density at different time points. **I ~ L** The peripapillary vessel density before and at 1,3,6 months after insulin intensification

During the follow-up period of 6 months after insulin intensification, the fundoscopy findings of all patients were stable, without diabetic retinopathy progression. At 1, 3, and 6 months after insulin intensification, both vessel density (VD) and retinal thickness in the macular area were lower than those before insulin intensification (Table 2, Fig. 1). Bonferroni’s post hoc test showed that both one month and six months after intensive insulin therapy, DCP-VD was lower than before treatment (*P* = 0.009, 0.000). Three months after intensive insulin therapy, SCP-VD, DCP-VD, and FD-300 were lower than those before intensification (*P* = 0.032, 0.000, and 0.039, respectively). In the oral hypoglycemic agent group, there were no significant differences in any macular parameter changes (all *P* > 0.05).

**Table 2:** Changes of vessel density (%), FAZ area (mm^2^), thickness (μm) in macular area in insulin intensive group (*n* = 36)

Macular Parameters	Pre-treatment	After treatment		
		1 M	3 M	6 M
SCP-VD	49.1 ± 2.8	48.0 ± 3.1	46.6 ± 2.7^*^	48.4 ± 3.1
DCP-VD	51.9 ± 3.9	47.8 ± 6.4^*^	45.9 ± 5.9^*^	46.1 ± 5.5^*^
FD-300	52.7 ± 4.0	50.3 ± 4.6	49.0 ± 4.7^*^	50.4 ± 4.2
FAZ area	0.28 ± 0.06	0.30 ± 0.05	0.31 ± 0.06	0.31 ± 0.06
Fovea thickness	251.7 ± 11.3	250.3 ± 11.0	248.4 ± 11.2	249.2 ± 10.9
Macular thickness	283.3 ± 17.4	282.4 ± 16.4	281.4 ± 14.4	282.9 ± 15.6

For the insulin intensive group, the significant changes compared with pre-treatment are marked with asterisks

*SCP* superficial capillary plexus; *DCP* deep capillary plexus; *FAZ* foveal avascular zone; *FD-300* foveal density in a 300μm wide region around FAZ; *1M* 1 month; *3M* 3 months; *6M* 6 months

**Fig. 2:**
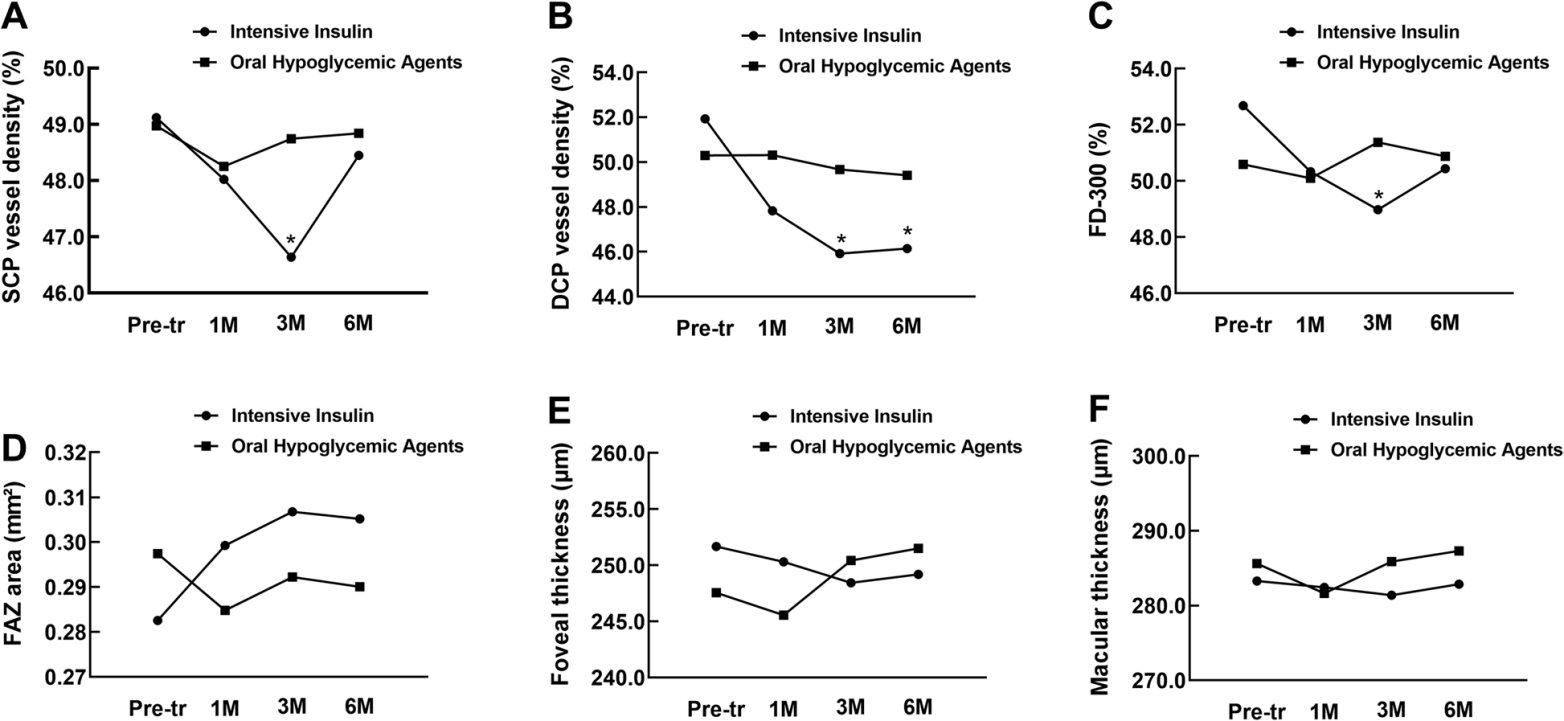
The longitudinal macular microvascular changes and macular thickness changes at pre-treatment, 1 M, 3 M, and 6 M following hypoglycemic treatment in the intensive insulin group and oral hypoglycemic agent group. The significant changes between the two groups compared at the same period points are marked with asterisks. **a** SCP vessel density changes; **b** DCP vessel density changes; **c** vessel density changes in FD-300; **d** FAZ area; **e** fovea thickness; **f** macular thickness. (Pre-tr = pre-treatment; 1 M = at 1 month follow-up)

Longitudinal macular changes in SCP-VD, DCP-VD, FD-300, and retinal thickness in the two groups are shown in Fig. 2. At three months after treatment, the vessel densities of SCP, DCP, and FD-300 in the intensification group were significantly lower than those in the oral agent’s group (*P* = 0.002, 0.004, and 0.039, respectively). Six months after treatment, DCP-VD in the intensification group was significantly lower than that in the oral agent group (*P* = 0.004).

In the intensification group, univariate analysis indicated a correlation between DCP-VD reduction and macular thickening at 1, 3, and 6 months after treatment (r = 0.348, *P* = 0.038; r = 0.693, *P *= 0.000 and r = 0.417, *P* = 0.011, respectively). No significant correlation was found between SCP-VD changes and macular thickness. Correlation between parameters of macular vessel density, FAZ area, and thickness changes in the insulin intensive group are shown in Table 3.

**Table 3:** Correlation between parameters of macular vessel density, FAZ area, and thickness changes in insulin intensive group (*n* = 36)

Parameters	SCP-VD		DCP-VD	
	*r*	*P* values	*r*	*P* values
FAZ area				
1 M	0.200	0.241	0.322	0.056
3 M	-0.281	0.097	0.035	0.841
6 M	-0.055	0.749	-0.253	0.137
Fovea thickness				
1 M	0.201	0.240	0.210	0.219
3 M	-0.086	0.620	0.224	0.189
6 M	0.053	0.759	-0.102	0.556
Macular thickness				
1 M	0.187	0.276	-0.348	0.038^‡^
3 M	0.147	0.391	-0.693	0.000^‡^
6 M	-0.190	0.266	-0.417	0.011^‡^

Statistically significant correlations are shown with ‡

### Changes in the peripapillary vessel density and corresponding RNFL thickness

After insulin intensification, ppVD and pRNFL were decreased at 1, 3, and 6 months compared with pre-treatment values (Table 4). According to the Bonferroni correction for multiple analyses, ppVD was lower at 1, 3, and 6 months after intensive insulin therapy than before treatment (*P* < 0.01). However, in the oral hypoglycemic agent group, there were no significant differences in the optic disc parameter changes (all *P* > 0.05).

**Table 4:** Changes of peripapillary vessel density (%), peripapillary RNFL thickness (μm) in insulin intensive group (*n* = 36)

Optic Disc Parameters	Pre-treatment	After treatment		
		1 M	3 M	6 M
ppVD	52.4 ± 3.3	47.9 ± 3.3^*^	48.6 ± 4.0^*^	49.0 ± 4.0^*^
pRNFL	112.4 ± 11.4	108.3 ± 12.1	107.9 ± 8.0	108.7 ± 11.0

The significant changes compared with pre-treatment are marked with asterisks

*ppVD* peripapillary vessel density; *pRNFL* peripapillary retinal nerve fiber layer

**Fig. 3:**
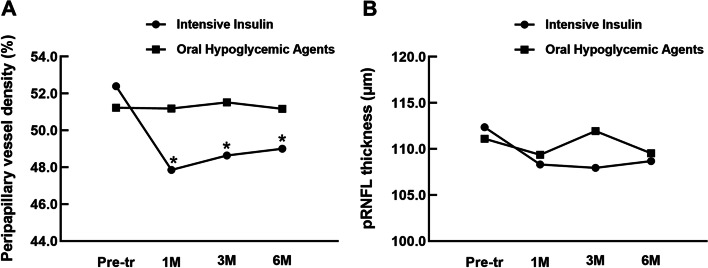
The longitudinal changes of ppVD and pRNFL at pre-treatment, 1 M, 3 M, and 6 M following hypoglycemic treatment in the intensive insulin group and oral hypoglycemic agent group. The significant changes between the two groups compared at the same period points are marked with asterisks. **a** ppVD changes; **b** pRNFL changes. (Pre-tr = pre-treatment; 1 M = at 1 month follow-up)

Figure 3 shows the longitudinal changes in PPVD and pRNFL in the intensive insulin therapy and oral hypoglycemic agent groups. During all follow-up periods, there were statistical differences in ppVD between the two groups (*P* = 0.000, 0.000, and 0.004, respectively).

## Discussion

In recent years, a growing number of imaging modalities have been used in the evaluation of DR. Although fundoscopy and fluorescein fundus angiography can intuitively and qualitatively evaluate the changes in retinopathy, they cannot reflect the changes in retinal density at different levels [17]. OCTA is non-invasive and can be repeatedly used to monitor changes in capillary levels [18]. Previous studies [19, 20] have demonstrated a decrease in retinal vessel density in preclinical diabetic retinopathy. Some studies have also shown [12, 21] that retinal capillary damage in early diabetic retinopathy does not entirely form a prominent non-perfusion area but represents decreased retinal vessel density. Thus, OCTA has a unique value for monitoring DR.

To the best of our knowledge, this study is the first to use OCTA to investigate the effects of insulin intensification on different retinal levels in patients with type 2 diabetes. A quantitative follow-up was conducted to observe the vessel density changes in the macular and optic disc areas and thickness parameters. To evaluate the degree of damage to the retinal capillary terminal, it is more accurate to detect changes in the microvascular structure before and after insulin treatment. We also noticed that the DCP-VD reduction was associated with macular thickening.

In our study, we found that insulin intensification caused decreased vessel density in the macular area, which initially reduced in the first month, significantly decreased at three months, and recovered at six months after treatment, but was still below the baseline level before intensification. In this study, three months after intensive insulin treatment, SCP, DCP, and FD-300 were significantly reduced after intensive insulin treatment, and the differences were statistically significant. Macular microvessel density was also statistically different from that of oral hypoglycemic agents during the same period. The results showed that insulin intensification caused the most severe damage to the microcirculation system of the superficial and deep capillaries of the retina at three months, leading to ischemic changes [22]. The decrease of macular microvasculature networks was most significant three months after intensification, but not earlier, and the reasons and mechanisms need to be further studied.

Interestingly, one month after intensification, the vessel densities of both SCP and FD-300 were slightly lower than those before treatment. However, the vessel density of the DCP group was significantly lower than that before treatment. Our findings showed that the early stage of intensive insulin therapy leads to adverse effects on the macular microvasculature, especially in the deep capillary plexus. One possible explanation for the preferential involvement of DCP ischemic changes is that DCP might be more sensitive to insulin intensification due to relatively lower blood flow in the deep capillary plexus [23, 24]. Histopathological studies have shown that diabetic micro hemangiomas originate mainly from the deeper capillary network [25], which indirectly proves that deep tissues are more susceptible to hypoxia. SCP mitigated hypoxic damage because it was directly connected to the retinal arterioles, which have a higher perfusion pressure and oxygen supply. These results are consistent with those of previous studies [26, 27]. Our study supports earlier results and adds new longitudinal evidence to previous cross-sectional observations regarding the unique value of microvascular changes in early DCP in diabetic patients [28].

It should be noted that, although the FAZ area increased after intensification, the difference was not statistically significant, considering that there is a considerable variation in the FAZ area of normal human eyes [29]. Our study added new longitudinal evidence to previous observations [30, 31] that the FAZ area of DR patients was more extensive than that of normal subjects. We also found that the thickness of the macular area during this period was also lower than that before intensification, indicating that structural damage may be related to the decrease in vessel density, but the difference in the sequence of intensification was not statistically significant.

The distinct pattern of microvascular changes in the intensification group brought us to consider whether there was an association between parameters of vessel density, FAZ area, and thickness of the macula, especially at the deep capillaries level. Previous studies have shown that diabetic patients with blurred vision after starting insulin therapy present a significant transient increase in macular biometrics [32]. In our study, changes of DCP-VD correlated negatively to the macular thickness at different time points after intensive insulin therapy.

From the perspective of longitudinal research, we found that retinal microcirculation was extensively affected after insulin intensification. The vessel density and thickness not only changed in the macular area but also decreased in the optic disc area. Microcirculation around the optic disc was the most obvious, and the radial peripapillary vessel density decreased significantly. It is speculated [33] that when vessel density declines in the macular area at the capillary terminal of the blood supply in ischemic retinopathy, ppVD upstream of the blood supply may also decrease. The results of this study are consistent with the above theoretical speculation; at one month after treatment, ppVD was statistically lower than that before treatment, while SCP-VD and FD-300 were not significantly changed until three months after treatment. This suggests that ppVD is similar to DCP-VD and reflects disease progression earlier than other indicators, suggesting that insulin intensification could cause microcirculation disorder of the optic disc area while early worsening of diabetic retinopathy (EWDR) progresses much earlier. Radial peripapillary capillaries are the innermost layer of capillaries that run parallel to the peripapillary RNFL and act as a nourish RNFL in its distribution around the optic nerve head. Frydkjaer-Olsen et al. [33] suggested that retinal vessel caliber was independently associated with structural changes of the neuroretina in patients with no or early DR prior to microangiopathy; however, there was no statistically significant decrease in peripapillary RNFL thickness after treatment, only presenting a trend of decline. A possible reason is that after ganglion cell injury, progressive dendrite contraction first occurs, followed by the disappearance of the cell body and axon; therefore, the reduction of ppVD may occur earlier than the thinning of RNFL thickness. Relevant literature indicates that compared with the regular control group, the decrease in ppVD in the whole week and each quadrant of the DM group was statistically different, while only part of the quadrants showed statistically significant differences in RNFL decline [34]. Therefore, it was speculated that the decrease of ppVD in DM patients might occur before the decline of RNFL, which is consistent with the results of this study. Monitoring RPC vessel density in the pericapillary region using OCTA may reveal neurodegeneration in the clinical stage of DR [35]. The addition of medications that improve retinal perfusion or optic nerve protection may slow the progression of optic nerve injury [36].

One might argue that a rapid decrease in blood glucose levels results in a transient reduction of retinal microcirculation. However, in the oral hypoglycemic agent group, there was no significant change in vessel density in the macular and optic disc areas after blood glucose decreased. This suggests that the decrease in vessel density was not due to fluctuations in blood glucose levels. The mechanism underlying decreased retinal vessel density after insulin intensification in patients with type 2 diabetes mellitus is unclear. Studies have shown [37–39] that insulin-like growth factor (IGF-1) can affect the function of retinal endothelial progenitor cells under hypoxia and promote retinal angiogenesis, which plays a vital role in the occurrence and development of DR and causes EWDR.

In the current study, we did not evaluate the relation between insulin doses and retinal vessel density. We could not draw a conclusion here because several difficulties existed in this investigation. First, patients in the intensive insulin group received different ways of intensification. Some patients were treated with insulin at least three times daily (fast-acting insulin + long-acting basal insulin), and some were treated using continuous subcutaneous insulin infusion (fast-acting insulins). Second, we use insulin lispro or insulin aspart for the fast-acting insulin, and insulin glargine, insulin detemir, or insulin degludec for the long-acting basal insulin. The pharmacodynamics of various insulin products show different effects on diabetic retinopathy. A 6-month phase 3 trial with insulin glargine versus human neutral protamine Hagedorn (NPH) insulin showed 7.0% versus 2.7% of patients in the insulin glargine versus NPH insulin group had DR progression [40]. However, this was disputed by the results of a 5-year trial investigating DR with insulin glargine versus NPH, which showed no difference in the rate of DR progression [41]. Third, the small sample size might be insufficient to yield significant results in correlation analysis. The relevance of insulin doses and diabetic retinopathy had been well demonstrated in previous studies. High-dose insulin might be one of the reasons for the transient worsening of diabetic retinopathy during intensive insulin treatment [42]. In the report by Reid et al. [43], CSII was associated with reduced diabetic retinopathy progression compared with continued MDI therapy, and maybe protect against diabetic retinopathy progression for those with high baseline HbA1c. Further studies are needed to explore the relationship between insulin doses and retinal vessel density.

There is no doubt that intensive treatment for glucose compliance can provide long-term benefits for most patients with type 2 diabetes [44]. However, retinal microvascular changes occur in the early stages of intensive treatment, indicating ischemia. The worsening of diabetic retinopathy manifests prior to the long-term benefits of optimizing glycemic control. Fundus conditions should be closely monitored in the early stage of intensive treatment.

The strengths of our study include its prospective study design, longitudinal OCTA follow-up, and high-quality data collection. This study has several limitations. First, the quality of the included samples was limited, so we could not determine the effect of different types of insulin on retinal microvascular density. Second, we only investigated the effects of intensive treatment on retinal microvessels, and choroidal vessel density should also be studied using more advanced OCTA machines. Third, only the macular (6.0 × 6.0 mm) and optic disc (4.5 × 4.5 mm) areas were involved, which could not reflect the blood perfusion measurement in a wider range, while the lesions site of DR could be located in the whole retina. Therefore, quantitative studies with a more extensive scanning range should be included in future studies [22, 45].

## Conclusions

During the 6-month follow-up period after insulin intensification, retinal vessel density decreased in the SCP, DCP, FD-300, and peripapillary capillaries. Our findings provide new insights into the changes in retinal microvessels before and after insulin intensification. Fundus conditions should be closely monitored during the early stage of insulin-intensive treatment. Monitoring of retinal microvasculature using OCTA is of great importance for patients with type 2 diabetes who are receiving intensive insulin therapy.

## Supplementary Information

**Additional file 1. **

## Data Availability

All data generated or analyzed during this study are included in this published article and its supplementary information files.

## Abbreviations

ANOVA: Analysis of variance; CHOL: Cholesterol;
CKD: Chronic kidney disease; DKD: diabetic kidney disease;
DBP: Diastolic blood pressure; DCP: Deep capillary plexus;
DME: Diabetic macular edema; DR: diabetic retinopathy;
eGFR: Estimated glomerular filtration rate; ETDRS: Early Treatment of Diabetic Retinopathy Study
FBG: Fast blood glucose; FAZ: foveal avascular zone;
FD-300: Foveal density in a 300 μm wide region around FAZ; HbA1c: glycated hemoglobulin A1c;
NDR: Without clinical DR; NPDR: non-proliferative diabetic retinopathy
ILM: Internal limiting membrane; SCP: Superficial capillary plexus;
OCTA: Optical coherence tomography angiography; OPL: outer plexiform layer;
PDR: Proliferative diabetic retinopathy; pRNFL: peripapillary retinal nerve fiber layer;
ppVD: Peripapillary capillaries vessel density; SBP: systolic blood pressure;
T2DM: Type 2 diabetes mellitus; TG: triglyceride;
VD: Vessel density; VEGF: vascular endothelial growth factor

## References

[1] Cheloni R, Gandolfi SA, Signorelli C (2019). Global prevalence of diabetic retinopathy: protocol for a systematic review and meta-analysis. BMJ Open.

[2] Qiu B, Zhao L, Zhang X (2021). Associations Between Diabetic Retinal Microvasculopathy and Neuronal Degeneration Assessed by Swept-Source OCT and OCT Angiography. Front Med Lausanne.

[3] Kramer CK, Zinman B, Retnakaran R (2013). Short-term intensive insulin therapy in type 2 diabetes mellitus: a systematic review and meta-analysis. Lancet Diabetes Endocrinol.

[4] Zoungas S, Arima H, Gerstein HC (2017). Effects of intensive glucose control on microvascular outcomes in patients with type 2 diabetes: a meta-analysis of individual participant data from randomised controlled trials. Lancet Diabetes Endocrinol.

[5] Pettitt DJ, Wollitzer AO, Jovanovic L (2005). Decreasing the risk of diabetic retinopathy in a study of case management. Diabetes Care.

[6] van B E, Hooymans J, immerman Z (1984). Rapid deterioration of diabetic retinopathy during treatment with continuous subcutaneous insulin infusion. Diabetes Care.

[7] Group TDCaCTR. Early worsening of diabetic retinopathy in the Diabetes Control and Complications Trial. Arch Ophthalmol. 1998;116:874–86 10.1001/archopht.116.7.874 9682700

[8] Feldman-Billard S, Larger É, Massin P (2018). Early worsening of diabetic retinopathy after rapid improvement of blood glucose control in patients with diabetes. Diabetes Metab.

[9] Bain SC, Klufas MA, Ho A (2019). Worsening of diabetic retinopathy with rapid improvement in systemic glucose control: A review. Diabetes Obes Metab.

[10] Li X, Yu Y, Liu X (2021). Quantitative analysis of retinal vessel density and thickness changes in diabetes mellitus evaluated using optical coherence tomography angiography: a cross-sectional study. BMC Ophthalmol.

[11] Mammo Z, Heisler M, Balaratnasingam C (2016). Quantitative Optical Coherence Tomography Angiography of Radial Peripapillary Capillaries in Glaucoma, Glaucoma Suspect, and Normal Eyes. Am J Ophthalmol.

[12] Sun Z, Yang D, Tang Z (2021). Optical coherence tomography angiography in diabetic retinopathy: an updated review. Eye (Lond).

[13] American DA (2014). Diagnosis and classification of diabetes mellitus. Diabetes Care.

[14] Ting DSW, Tan GSW, Agrawal R (2017). Optical Coherence Tomographic Angiography in Type 2 Diabetes and Diabetic Retinopathy. JAMA Ophthalmology..

[15] Wilkinson CP, Ferris FL, Klein RE (2003). Proposed international clinical diabetic retinopathy and diabetic macular edema disease severity scales. Ophthalmology.

[16] Umpierrez GE, Hellman R, Korytkowski MT (2012). Management of hyperglycemia in hospitalized patients in non-critical care setting: an endocrine society clinical practice guideline. J Clin Endocrinol Metab.

[17] Li X, Xie J, Zhang L (2020). Differential distribution of manifest lesions in diabetic retinopathy by fundus fluorescein angiography and fundus photography. BMC Ophthalmol.

[18] Spaide RF, Fujimoto JG, Waheed NK (2018). Optical coherence tomography angiography. Prog Retin Eye Res.

[19] Cao D, Yang D, Huang Z (2018). Optical coherence tomography angiography discerns preclinical diabetic retinopathy in eyes of patients with type 2 diabetes without clinical diabetic retinopathy. Acta Diabetol.

[20] Dimitrova G, Chihara E, Takahashi H (2017). Quantitative Retinal Optical Coherence Tomography Angiography in Patients With Diabetes Without Diabetic Retinopathy. Invest Ophthalmol Vis Sci.

[21] Nesper PL, Roberts PK, Onishi AC, et al. Quantifying Microvascular Abnormalities With Increasing Severity of Diabetic Retinopathy Using Optical Coherence Tomography Angiography. Invest Ophthalmol Vis Sci. 2017;58:BIO307–15. 10.1167/iovs.17-21787 PMC5693005 29059262

[22] Lam PY, Chow SC, Lam WC (2021). Management of Patients with Newly Diagnosed Diabetic Mellitus: Ophthalmologic Outcomes in Intensive versus Conventional Glycemic Control. Clin Ophthalmol.

[23] Sun Z, Tang F, Wong R (2019). OCT Angiography Metrics Predict Progression of Diabetic Retinopathy and Development of Diabetic Macular Edema: A Prospective Study. Ophthalmology.

[24] Kim AY, Chu Z, Shahidzadeh A (2016). Quantifying Microvascular Density and Morphology in Diabetic Retinopathy Using Spectral-Domain Optical Coherence Tomography Angiography. Invest Ophthalmol Vis Sci.

[25] Horii T, Murakami T, Nishijima K (2010). Optical coherence tomographic characteristics of microaneurysms in diabetic retinopathy. Am J Ophthalmol.

[26] Simonett JM, Scarinci F, Picconi F (2017). Early microvascular retinal changes in optical coherence tomography angiography in patients with type 1 diabetes mellitus. Acta Ophthalmol.

[27] Onishi AC, Nesper PL, Roberts PK (2018). Importance of Considering the Middle Capillary Plexus on OCT Angiography in Diabetic Retinopathy. Invest Ophthalmol Vis Sci.

[28] Karst S, Salas M, Hafner J (2019). Three dimensional analysis of retinal microaneurysms. Retina.

[29] Yu J, Jiang C, Wang X (2015). Macular perfusion in healthy Chinese: an optical coherence tomography angiogram study. Invest Ophthalmol Vis Sci.

[30] Di G, Weihong Y, Xiao Z (2016). A morphological study of the foveal avascular zone in patients with diabetes mellitus using optical coherence tomography angiography. Graefes Arch Clin Exp Ophthalmol.

[31] Attia Ali Ahmed M, ShawkatAbdelhaleem A (2022). Evaluation of Microvascular and Visual Acuity Changes in Patients with Early Diabetic Retinopathy: Optical Coherence Tomography Angiography Study. Clin Ophthalmol.

[32] Hernandez C, Zapata MA, Losada E (2010). Effect of intensive insulin therapy on macular biometrics, plasma VEGF and its soluble receptor in newly diagnosed diabetic patients. Diabetes Metab Res Rev.

[33] Frydkjaer-Olsen U, Soegaard Hansen R, Simo R (2016). Correlation between Retinal Vessel Calibre and Neurodegeneration in Patients with Type 2 Diabetes Mellitus in the European Consortium for the Early Treatment of Diabetic Retinopathy (EUROCONDOR). Ophthalmic Res.

[34] Cao D, Yang D, Yu H (2019). Optic nerve head perfusion changes preceding peripapillary retinal nerve fibre layer thinning in preclinical diabetic retinopathy. Clin Exp Ophthalmol.

[35] Cheung CY, Chen D, Wong TY (2011). Determinants of quantitative optic nerve measurements using spectral domain optical coherence tomography in a population-based sample of non-glaucomatous subjects. Invest Ophthalmol Vis Sci.

[36] Hernandez C, Simo R (2012). Neuroprotection in diabetic retinopathy. Curr Diab Rep.

[37] Raman P, Singal AK, Behl A (2019). Effect of Insulin-Like Growth Factor-1 on Diabetic Retinopathy in Pubertal Age Patients With Type 1 Diabetes. Asia Pac J Ophthalmol (Phila).

[38] Kondo T, Vicent D, Suzuma K (2003). Knockout of insulin and IGF-1 receptors on vascular endothelial cells protects against retinal neovascularization. J Clin Investig.

[39] Chantelau E, Meyer-Schwickerath R, Klabe K (2010). Downregulation of serum IGF-1 for treatment of early worsening of diabetic retinopathy: a long-term follow-up of two cases. Ophthalmologica.

[40] Rosenstock J SS, Clark CM Jr, Park GD, Donley DW, Edwards MB. Basal insulin therapy in type 2 diabetes: 28-week comparison of insulin glargine HOE 901 and NPH insulin. Diabetes Care. 2001;24:631–6. 10.2337/diacare.24.4.631 11315821

[41] Rosenstock J, Fonseca V, McGill JB (2009). Similar progression of diabetic retinopathy with insulin glargine and neutral protamine Hagedorn (NPH) insulin in patients with type 2 diabetes: a long-term, randomised, open-label study. Diabetologia.

[42] Wu H, Jiang C, Gan D (2011). Different effects of low- and high-dose insulin on ROS production and VEGF expression in bovine retinal microvascular endothelial cells in the presence of high glucose. Graefes Arch Clin Exp Ophthalmol.

[43] Reid LJ, Gibb FW, Colhoun H (2021). Continuous subcutaneous insulin infusion therapy is associated with reduced retinopathy progression compared with multiple daily injections of insulin. Diabetologia.

[44] Li Y, Xu W, Liao Z (2004). Induction of long-term glycemic control in newly diagnosed type 2 diabetic patients is associated with improvement of beta-cell function. Diabetes Care.

[45] Barrett EJ, Liu Z, Khamaisi M (2017). Diabetic Microvascular Disease: An Endocrine Society Scientific Statement. J Clin Endocrinol Metab.

